# First Outbreak of African Swine Fever in Sweden: Local Epidemiology, Surveillance, and Eradication Strategies

**DOI:** 10.1155/2024/6071781

**Published:** 2024-06-26

**Authors:** Erika Chenais, Viktor Ahlberg, Kristofer Andersson, Fereshteh Banihashem, Lars Björk, Maria Cedersmyg, Linda Ernholm, Jenny Frössling, Wiktor Gustafsson, Lena Hellqvist Björnerot, Cecilia Hultén, Hyeyoung Kim, Mikael Leijon, Anders Lindström, Lihong Liu, Anders Nilsson, Maria Nöremark, Karin M. Olofsson, Emelie Pettersson, Thomas Rosendal, Marie Sjölund, Henrik Thurfjell, Stefan Widgren, Emil Wikström-Lassa, Siamak Zohari, Erik Ågren, Estelle Ågren, Karl Ståhl

**Affiliations:** ^1^ Swedish Veterinary Agency, SVA, Uppsala 75189, Sweden; ^2^ Swedish University of Agricultural Sciences, SLU, Box, 7070, Uppsala 75007, Sweden; ^3^ Swedish Association for Hunting and Wildlife Management Svenska Jägareförbundet, Öster Malma, Nyköping 61191, Sweden; ^4^ Swedish Board of Agriculture Jordbruksveket, Jönköping 551 82, Sweden

## Abstract

The first case of African swine fever (ASF) was confirmed in Sweden in September 2023. This article describes the local epidemiology, including the spatiotemporal dynamics of the outbreak and some of the factors that may have contributed to its apparently successful eradication. Upon detection of the outbreak, strict control measures were put in place in a preliminarily defined infected zone. A carcass search, including geo-localisation, removal, sampling, and destruction of found carcasses, was initiated and a preliminary core area was defined based on the results. Six months after confirmation of the first case, 93 wild boar carcasses had been found in the infected zone, of which 62 tested positive for ASF virus (ASFV). All ASFV-positive carcasses were found inside the core area. Based on two taphonomy methods, it was assumed that the infection was introduced between early May and late June 2023. The data also indicated that the epidemic curve peaked between mid-August and mid-September, with the last death occurring in late September 2023. Based on the average estimated time of death, geo-localisation of carcasses and two-dimensional kernel density estimation, clustering in space and time was identified. An online questionnaire with questions about hunting and the wild boar population was sent to all leaders of hunting groups in the infected zone. The results showed that the wild boar population had increased in the last 10 years but with large variations and geographical heterogeneity in space use. Disease introduction through natural wild boar movements was excluded and it was assumed that the long-distance translocation of the virus had occurred through human activities. A municipal waste collection centre without wild boar-proof fencing is located close to the epicentre of the outbreak, attracting many wild boar and contributing to the spread of the virus once it had been introduced to the population.

## 1. Introduction

In September 2023, African swine fever (ASF) was confirmed in wild boar in Sweden. This was the first time that the ASF virus (ASFV) had been detected in the country, making Sweden part of the current global ASF epidemic that started in Georgia in 2007 [1]. In northern and central Europe, the epidemic has primarily affected wild boar with some spill-over to domestic pigs but very limited spread between pig holdings [2, 3]. In southern and south-eastern Europe, a different scenario can be seen, with the epidemic mainly affecting domestic pigs in backyard farms and spilling over to wild boar populations [4, 5]. As with domestic pigs, ASFV infection in wild boar typically results in severe clinical disease with high case fatality rates [6, 7, 8]. Controlling ASF in wild boar populations has proved difficult. In the current epidemic, so far only Belgium and the Czech Republic have managed to eradicate the disease after it had been introduced into a wild boar population (the Czech Republic was re-infected in 2022) [9, 10]. In both these cases, the disease is assumed to have been introduced via long-distance human translocation, followed by point introductions into the local wild boar populations.

The risk of ASFV being introduced in Sweden has repeatedly been assessed as “increased, but at a low level” (level 3 out of 6, using a terminology for the grading of probability ranging from negligible to very high) in the 10 years since ASFV was first detected in the European Union (EU). A human-mediated long-distance translocation resulting in point introduction into the wild boar population in one of the counties in southern Sweden with a higher wild boar density has been considered the most probable scenario (https://www.sva.se/media/8d934c1b8d527ec/yttrande-riskkartlaggning-asf.pdf).

This article aims to describe the outbreak of ASF in Sweden, with a specific focus on the local epidemiology and how the responsible authorities organised their work in the first few months. Sharing these experiences might serve to guide ASF-free countries in their contingency planning, and shed light on some of the questions that still remain concerning the epidemiology of ASF in the wild boar-habitat cycle [3].

### 1.1. Wild Boar Population in Sweden and in the Infected Zone

**Figure 1:**
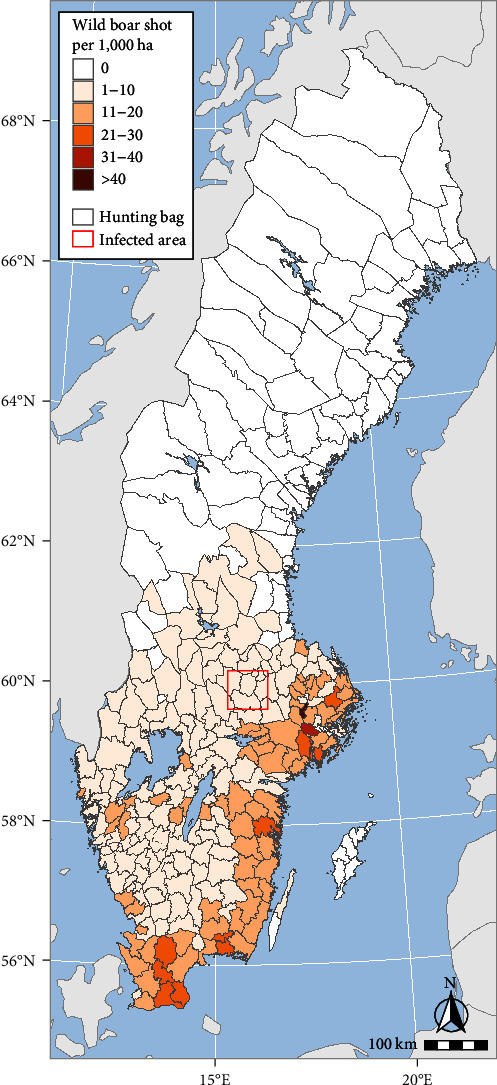
Wild boar hunting bag in Sweden in 2022, expressed as the number of wild boar shot per 1,000 hectares. Darker colours indicate more wild boar shot. The approximate area of the outbreak of African swine fever is marked with a square.

Based on the national hunting bag, traffic accidents with wild boar and the number of wild boar tested for *Trichinella suis*, the Swedish wild boar population was estimated to be approximately 300,000 in the 2021–2022 hunting year. In Sweden, as in most other European countries, the reporting of shot wild boar or any other reporting of wild boar abundance is not obligatory. This makes wild boar population density estimations imprecise [11, 12]. The wild boar population in Sweden is unevenly distributed, with higher densities in the south and south-east and almost no wild boar north of latitude 62°N (Figure 1). The population size and the areas populated by wild boar have increased every year from the late twentieth century up to 2022. In the last two hunting years, however, an apparent decrease has been observed. In the climatic and vegetation conditions that prevail in Sweden, the severity of winter is the limiting factor [13], although this can be offset by access to supplemental feeding [14]. Baiting is frequently used to boost hunting success, and supplemental feeding has been used to increase the local population or redirect wild boar away from roads or crops.

In the 2021–2022 hunting year, 316 wild boar were reported shot in the six administrative hunting areas included in the ASF-infected zone as established on 7 September 2023 (see “The outbreak”), i.e., less than 0.5 wild boar shot per kilometer square, suggesting a relatively low density.

## 2. The Outbreak

**Figure 2:**
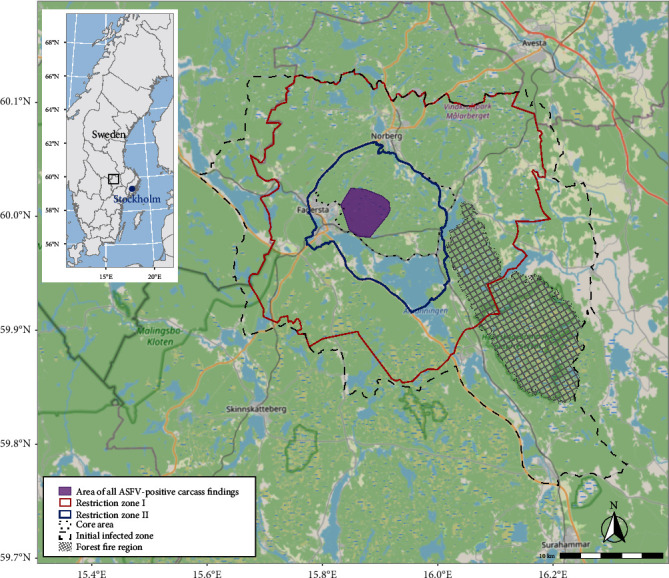
A map indicating the location of the ASF outbreak in Sweden. The dashed line marks the infected zone defined on 7 September 2023, the dotted line marks the core area of the outbreak, and the red and blue lines mark the zones established by the European Commission on 30 November 2023 (red line = restricted zone I, blue line = restricted zone II, fenced off). The purple area marks the area in which all the infected carcasses were found. The crosshatched zone marks an area that was affected by wildfire in 2014 that could not easily be accessed by foot.

On 25 August 2023, a hunter in Fagersta municipality, approximately 170 km north-west of Stockholm, reported two findings of wild boar on his hunting ground (see Figure 2 and Supplementary Material). One of the wild boar was found alive, but was immobile and so was euthanised; the other was found dead. This was reported using the online reporting form (“Rapportera vilt”, rapporteravilt.sva.se), which is in regular use for Sweden's wildlife disease surveillance programme managed by the Swedish Veterinary Agency (SVA). Neither carcass could be located when SVA requested that samples be taken and submitted. On 28 August, a third wild boar carcass was reported from the same hunting ground, and SVA sent sampling and transport material to the reporting hunter. The distal part of a front leg was removed for sampling and sent to SVA on 4 September. The package arrived at SVA on 5 September, and a sample of bone marrow was taken for ASFV analysis. On 6 September, ASFV was detected by real-time PCR in the bone marrow from the submitted sample. To clarify the situation and avoid repeated reporting and sampling of the same carcasses, at that point in time the local hunters were asked to draw a map showing where all the wild boar carcasses had been found and to send this information to SVA. The map showed a total of six wild boar carcasses and one sick, subsequently euthanised wild boar, with the longest distance between any two carcasses being 3 km. The carcasses were described as being in varying degrees of putrefaction.

On 7 September, the Swedish Board of Agriculture (SBA) declared an area of approximately 1,000 km^2^ around Fagersta municipality as a so-called “infected zone” in accordance with the EU's animal health legislation (EU 2016/429) (Figure 2). In this zone, a set of restrictions was put in place to contain and eradicate the outbreak, with the ultimate aim to regain freedom from ASF in Sweden. The infected zone was defined considering ASF epidemiology in wild boar, the local wild boar population including spatial continuity and habitat, as well as natural borders (roads, water, game fences along major roadways) and administrative borders. Large parts of the infected zone, as well as the areas west and north of it, comprise nutrient-poor boreal forests typical for the area, which are not favourable habitats for wild boar.

An active search for carcasses started in the infected zone on 9 September 2023. Based on the search and test results, and taking into consideration natural borders for wild boar movements, a 100-km^2^ core area of the outbreak was identified on 14 September 2023. With effect from 30 November, the infected zone was replaced by a so-called restricted zone, encompassing restricted zones I and II (RZ I and RZ II) (RZ I = an area bordering a location with ASF outbreaks, RZ II = an area with an outbreak of ASF in wild porcine animals.) in accordance with the EU legislation (EU 2023/594). Based on a risk assessment including factors such as the control actions taken and the positive evolution of the outbreak (see the Results section), the total area under restriction (RZ I + RZ II) could at this stage be reduced in comparison with the initial infected zone, and now encompassed 618 km^2^. RZ II (in total 148 km^2^) included the core area and was fenced off (Figure 2).

### 2.1. Control Actions

To facilitate outbreak management, the SBA set up a local disease control centre and a sampling centre (including a mobile incinerator) at the outbreak location, which was in operation from 9 September 2023.

The actions and control measures taken were based on knowledge about ASF epidemiology in wild boar, local conditions, and previous experience of ASF in Europe, including control of single-point introductions of ASFV [10, 15] as well as recommendations from the EFSA [16]. Restrictions included an initial ban on all activities in forests and other rural areas apart from gardens, agricultural land, roads, and established sports arenas in the infected zone. The purpose of the restrictions was to prevent indirect spread of the infection within or outside the infected zone via people, vehicles, and materials, and to reduce the risk of disturbing wild boar away from their established home ranges within the infected zone. To reduce wild boar movements further, existing baiting sites within the infected zone were continuously maintained, and some additional baiting sites were established. For the same purpose, some crop fields within the infected zone were left unharvested, with farmers compensated for the financial loss by SBA. A municipal waste collection centre without wild boar-proof fencing was located in the core area, close to where all the infected carcasses were found. For several years, wild boar had been known to frequent the site in search of food in the residual waste fraction (including household waste, public waste bins, and bins from roadside rest areas) that was left openly exposed overnight. To reduce wild boar movements, the residual waste fraction was initially kept accessible for wild boar. During this time, the site was closed for public access and biosecurity routines were introduced to prevent ASFV spread from the site.

Only six domestic pig holdings with a total number of 59 pigs, five of which were classified as backyard holdings and one as commercial, were located in the infected zone. As a preventive measure, all these pigs were culled within the second week of the outbreak.

As the positive evolution of the outbreak allowed for the outbreak area to be reduced upon its modification into RZ I and RZ II, the restrictions were adapted accordingly. The same strict ban on all activities in forests and other rural areas was kept in RZ II, while restrictions were reduced in RZ I, including granting the general public access to forests and rural land. Organised events with large groups of people, hunting, the use of motor-driven vehicles off roads, and forestry activities continued to be banned in RZ I. Exceptions could be granted upon application to, and approval from, the SBA. Changes in restrictions were communicated through press conferences and press releases. Local meetings were repeatedly held in all affected municipalities with the participation of disease experts and decision-makers from SVA and SBA.

A decision was taken to construct a fence to reduce wild boar migration in and out of the core area. For practical reasons (existing game fences) and to allow a sufficiently large buffer between the positive cases and the fence, the fenced-in area was larger than the identified core area (148 versus 100 km^2^). Fencing activity started on 11 October 2023. The fence was a 1.2-m-high knotted wire mesh with secured nonslip knots and wooden poles. The poles were substantially higher than the fence to prepare for the installation of a complementary electrical wire in the event of deep snow cover in winter that could effectively render the fence too low to prevent wild boar from passing over it. On the side facing the core area, the lower part of the fence was reinforced with a second net that was angled out flat onto the ground to prevent wild boar from lifting the bottom of the fence to pass underneath. Almost half of the perimeter of the core area was already delimited by permanent double game fences along major roadways. These were reinforced with the same type of ground-covering net as the newly installed fence. Wildlife underpasses along one of the roads with game fencing were closed off with wire mesh fences. At junctions with major roads leading into the core area, the fence followed the connecting road for at least 50 m. Smaller roads were sealed with gates. As the fence crossed railways, both wooden fences and so-called “pyramid rubber mats” (http://trafikverket.diva-portal.org/smash/get/diva2:1365301/FULLTEXT01.pdf) designed to prevent crossing were used, as well as a motion sensor-operated wildlife-deterring sound device to deter wild boar. The full perimeter of the fence was patrolled once a week to check for damage and signs of wild boar crossing. No instances of wild boar passing under or jumping over the fence were reported.

Culling of wild boar inside and close to the core area started as soon as the fence had been completed on 22 November 2023. The goal was to eliminate all remaining wild boar in RZ II, and then keep this zone free of wild boar. Culling in corral traps (https://pigbrig.com and a plywood version of the trap described in Fahlman et al. [17]) and shooting at baiting sites were undertaken without inducing the movement of wild boar. All culled wild boar were subjected to testing and the carcasses were incinerated.

### 2.2. Multi-Stakeholder Collaboration

In Sweden, SBA is the competent authority responsible for disease control and risk management. SVA is the expert authority responsible for risk assessment, and it advises SBA on epidemiology and provides national veterinary reference laboratory facilities for ASF and all other diseases regulated by Swedish or European law. These two authorities cooperate regularly on animal disease control and surveillance. In outbreaks of infectious animal diseases that affect wildlife, multi-stakeholder collaboration is necessary [18]. Apart from the stakeholders regularly involved in animal disease outbreaks and control (such as central veterinary authorities, field veterinarians, local and regional authorities, farmers and farmers' organisations), previous experience of ASF in Europe has highlighted the importance of participation, collaboration, and communication with, among others, local hunters, hunters' organisations, landowners, and environmental authorities [19, 20, 21]. In Sweden, a collaboration of this kind had been established prior to the outbreak and it intensified from the first day of the outbreak. For example, regular communication meetings have been held since 2019 with a broad stakeholder group consisting of around 60 different entities, such as central, regional and local authorities, veterinary health providers, the pig and pork industry, as well as hunters, farmers, forestry industry, landowners, and sports and recreation associations. Furthermore, a previously formed expert group at SVA, consisting of experts from SVA on ASF epidemiology, wildlife and pig health, a wild boar ecology expert from the Swedish University of Agricultural Sciences (SLU) and a representative from the Swedish Association for Hunting and Wildlife Management (SJF), became operational immediately upon disease confirmation. SJF represents about 55% of all registered hunters in Sweden and has local associations in all counties and an efficient network of staff and trustees that can reach out to its members with information or requests for voluntary participation. To ensure local knowledge about hunting and the wild boar population in the outbreak area, a representative from SJF's regional office was included in the expert group.

## 3. Materials and Methods

### 3.1. Carcass Search

An active search for carcasses was initiated to determine the true extent of the outbreak area and establish its core area. To reduce the risk of disease spread, all found carcasses, or parts thereof, were removed from the forests. The carcass search was done by foot, initiated by SVA together with SBA and the local disease control centre, and coordinated by SJF. The majority of the people performing the searches were local hunters, but other volunteers from the local community also participated. Dogs on the lead were allowed, and used to a limited extent, in the searches. Searches were initially performed on a voluntary basis and later with remuneration for the work (including retroactive compensation for the initial voluntary work). At first, a “from-the-centre-and-out” strategy was applied, with the six first reported carcasses as the central point. In addition, habitat preferred by wild boar was prioritised for the initial searches, with hunters primarily searching their own hunting grounds where they were very familiar with the terrain, the wild boar population, and the preferred rest areas for wild boar. Later, search priorities were set weekly, based on previously unsearched areas (with the aim of covering the entire infected zone) and previous carcass findings. Biosecurity training and registration were compulsory for participation in the search and to receive remuneration. Equipment for cleaning footwear and personal equipment after the search was provided. Search areas were allocated in morning meetings on every search day, with two to three people searching together to cover a square area. This search pattern was chosen to cover the ground but disturb live wild boar less than if the search had been organised in the form of a linear search party. The searchers recorded their search path in the WeHunt® mobile application. An agreement between WeHunt® and SVA allowed for the search patterns to be collected automatically and digitally transferred to SVA daily. Before this system was fully functional, the areas covered in the searches were drawn manually on maps. To complement this, the search leader for each area also used another freely available mobile application, Gaia GPS (https://www.gaiagps.com/), to draw a map before emailing it as a GPX file to SVA. At SVA, the searched areas were digitally compiled, analysed, visualised, and used to monitor the search progress and set new search priorities. Land-cover data were retrieved from the Swedish Land Survey authority and used to analyse the parts of the zone that were searchable on foot.

Carcass findings were registered, given a unique identifier, photographed, and geo-localised by the searchers using a web application developed specifically for the outbreak (based on the existing public “Rapportera vilt” form). Following a report in the web application, a carcass patrol was sent out to retrieve the carcass using all-terrain vehicles and/or plastic sled or plastic bags, and considering strict biosecurity routines including personal protective equipment as well as cleaning and disinfection of vehicles and equipment at the return to the sampling centre. Carcasses were brought to the sampling centre, sampled (spleen or a long bone) by an official veterinarian, and incinerated in a mobile incinerator (Hurrikan 500, Waste Spectrum Ltd., Worcestershire, UK). The decomposition status of the majority of carcasses was noted using a protocol adapted from Probst et al. [22] (Supplementary Materials). The form was completed by the person who found the carcass, the person who collected it or the person who performed the sampling. From 21 November 2023, the assessment was done using a web-based form. Samples were transported to SVA and generally analysed the next day. The unique carcass identifiers allowed samples to be digitally connected to their corresponding carcasses and their metadata, which facilitated quality control and the timely communication of results to the public (https://www.sva.se/en/what-we-do/contagion-status/surveillance-of-african-swine-fever-asf/monitoring-of-african-swine-fever-asf/).

### 3.2. Surveillance

In the infected zone, all the carcasses found during the carcass search, all the wild boar killed by traffic and all culled wild boar were sampled at the sampling centre. Samples were transported to SVA and tested for ASFV. Furthermore, enhanced surveillance in an area encompassing all the municipalities surrounding the infected zone was initiated on 7 October 2023. This included enhanced passive surveillance of wild boar in the form of voluntary sampling of hunted wild boar and sampling of all wild boar found dead or killed by traffic. Moreover, systematic surveillance of domestic pigs was implemented based on the testing of two dead pigs per week in all holdings with more than 250 pigs. In holdings with fewer than 250 pigs, a veterinary assessment was to be undertaken of any pig death to decide if the death raised a suspicion of ASF or establish whether the pig was suitable for routine passive surveillance. The area included in the enhanced surveillance was adapted according to changes in the outline of the zone, with the same measures applied in the zones and municipalities surrounding the respective zone all the time.

### 3.3. Laboratory Analysis

Detection of ASFV was performed at SVA using real-time PCR. Samples were extracted using an IndiMag Pathogen Kit (Indical Bioscience, Leipzig, Germany) on a Maelstrom 9600 (TANBead, Taoyuan City, Taiwan) nucleic acid extraction robot. PCR was performed using PerfeCTa qPCR ToughMix (Quantabio, Beverly, MA, USA) with primers described by Fernández-Pinero et al. [23] and a probe in accordance with the WOAH Terrestrial Manual [24]. In addition, samples were sent for confirmation to the European reference laboratory for ASF (Centro de Investigacion en Sanidad Animal (INIA-CISA/CSIC)) and to the Friedrich Loeffler Institute (FLI).

In an effort to improve understanding of the origin of the virus, whole-genome sequencing (WGS) was performed. A positive sample from one of the initial cases with a relatively low Ct value of 19 was selected and subjected to metagenomic next-generation sequencing (NGS) using an Illumina MiSeq instrument (Illumina Inc., San Diego, CA, USA) [25]. Library construction was performed using a NEXTERA-XT kit (Illumina Inc., San Diego, CA, USA) according to the manufacturer's instructions. The quality of the libraries obtained was assessed by the Agilent 2100 Bioanalyzer (Agilent Technologies, Santa Clara, CA, USA). Libraries were sequenced on a MiSeq Instrument (Illumina Inc., San Diego, CA, USA), using a Miseq Reagent Kit v3 in a 600-cycle paired-end run. Generated data were analysed using the CLC genomics workbench v21 (CLC bio, Aarhus, Denmark). Low-quality reads and adaptor sequences were removed prior to mapping against a closely related sequence 20355/RM/2022_Italy (OP605386) and the consensus sequence of all available ASFV complete genome sequences at GenBank. Deletions, insertions, and unique point mutations in CVR, IGR I73R/I329L, O174L, K145R, IGR MGF5059R/10R, and ECO21329L1215L regions were used for further characterisation of the sequence, as described by Gallardo et al. [26], to assign the sequence to one of the 25 genetic groups currently identified within the genotype II-ASFVs.

### 3.4. Spatiotemporal Epidemiology of the Outbreak

**Figure 3:**
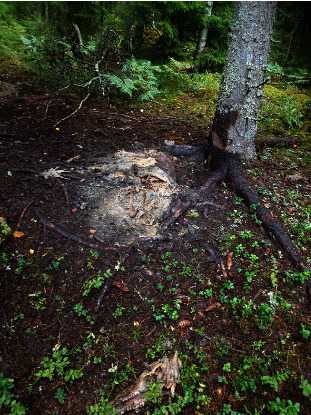
The carcass used to estimate time of death based on the taphonomy model [27] in the outbreak of African swine fever in wild boar in Sweden in 2023.

A taphonomy model, developed and evaluated for humans, was used to estimate the time of death of a selected carcass [27]. The carcass deemed to be the most representative for determining the time of introduction out of the ASFV-positive carcasses found up to 5 October 2023 (based on available photographs) was selected for analysis (Figure 3). This carcass was found on 6 September 2023 and confirmed positive for ASFV on 13 September 2023. Briefly, the method involves rating the decomposition state of the carcass, and inserting the score into a formula that gives the number of accumulated day degrees (ADD) needed to reach that stage of decomposition. The formula is ADD = 10 ^(0.002^*∗*^TBS ^*∗*^TBS + 1.81)^ ± 388.16, where TBS stands for total body score, the sum of the decomposition score from a table in the paper. Temperature data were retrieved from the Swedish Metrological and Hydrological Institute (SMHI), using the closest station (Sala A), where current temperatures were assumed to be representative of the area in which the carcass was found, allowing the calculation of a timespan for the death of the wild boar. This model was chosen in the absence of corresponding models for determining the time of death of animals, under the assumption that it is also applicable to wild boar carcasses. Given that domestic pig carcasses are often used to study human decomposition, this was considered a fair assumption.

**Figure 4:**
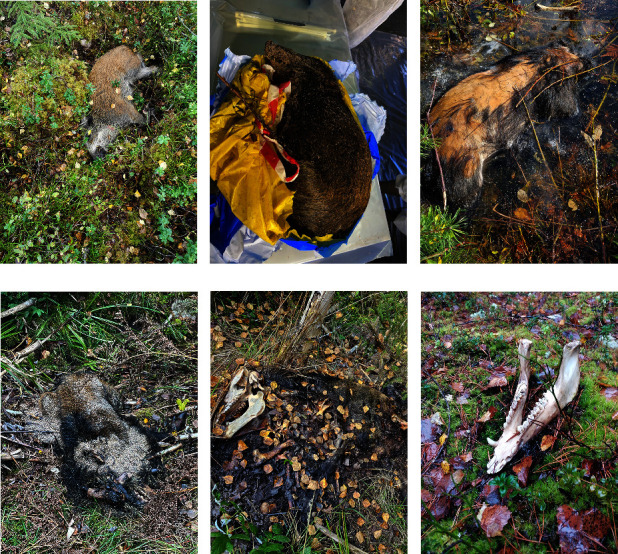
(a–f) The six stages of decay exemplified by photographs of wild boar found in the core area of the outbreak of African swine fever in wild boar in Sweden in 2023: (a) fresh, (b) early decay (photograph from the sampling centre), (c) bloated, (d) post-bloated, (e) advanced decay, and (f) dry remains.

In addition to this detailed analysis aiming to determine the time of introduction, a faster pathologic evaluation of available photographs and/or carcass protocols from all ASFV-positive carcasses found up to 20 November was performed to estimate the time of death for each carcass. Based on a previous publication on wild boar carcass decomposition for a habitat similar to that of the affected area in Sweden[28], six stages of decay were set with a corresponding interval for the estimated time between the death and the finding of the carcass (Table 1). Examples of each category are given in Figures 4(a), 4(b), 4(c), 4(d), 4(e), and 4(f). During the evaluation, photographs and/or the carcass status protocol were examined in parallel by two wildlife pathologists. For each carcass, the time of death was stated as an interval and as the average of that interval. This screening was done to complement the taphonomy model assessment regarding the time of introduction and create an epidemiological overview of the temporality of the outbreak.

**Figure 5:**
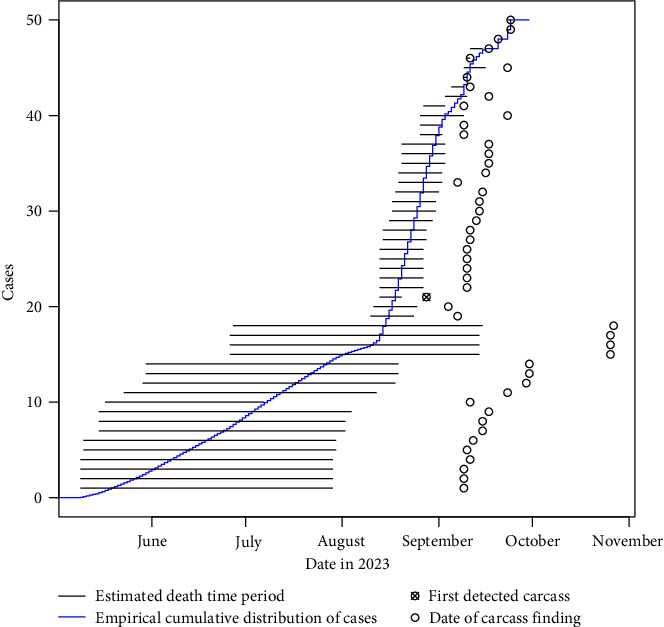
Time of death estimation of 50 carcasses positive for African swine fever (ASF) found between 25 August and 30 November 2023 in the outbreak of ASF in wild boar in Sweden. The estimated death time interval of each carcass (based on a photographic pathologic evaluation adopted from Rietz et al. [28]) is represented by a horizontal line and the date of detection is indicated by a circle. The first detected carcass is indicated by a crossed-over circle. The carcasses are sorted by the first date of the estimated interval. The blue line represents the empirical cumulative distribution of the estimated death times, and the slope of the curve represents the rate of the epidemic growth.

A uniform distribution of the daily probability of death was assumed in the estimated time interval for each carcass. Based on this, an empirical cumulative epidemic curve was constructed with the sum of the daily probabilities from all carcasses (Figure 5). Using the geocoordinates indicating the location of each carcass and the average time of death, the spatiotemporal evolution of the outbreak was plotted on maps using a two-dimensional kernel density estimation with a 1,000 m bandwidth [29].

### 3.5. Wild Boar Population and Management

To assess the local wild boar abundance before and during the outbreak, an online questionnaire with questions concerning past and present observations of wild boar, hunting ground parameters, hunting bags, trail cameras, and wild boar baiting was compiled using an online software (Questback, Stockholm, Sweden) (Supplementary Materials). The questionnaire was distributed via an e-mail sent by the local representative of SJF to contact persons for each of the hunting grounds (*n* = 120) in the infected zone on 27 November 2023. Contact details were extracted from SJF's hunting management and reporting system (viltdata.se). The respondents were instructed to send one reply per hunting ground (*n* = 96). A reminder was sent out on 12 December 2023 and the questionnaire was closed on 13 February 2024.

## 4. Results

### 4.1. Carcass Search

Out of the 1,000 km^2^ in the initial infected zone, a 115 km^2^ area had been affected by a wild forest fire in 2014. Large parts of this area could not be accessed on foot easily or safely. Water was excluded from the searchable area, although a search of lakeshores was undertaken by boat. Urban areas and other areas with buildings were not included in the searches. An area of 774 km^2^ belonging to a landscape cover category that was deemed searchable on foot was used to monitor the search process (“coniferous and mixed forest”, “deciduous forests”, “open land”, and “arable land”). Between 9 September and 30 November, 629 km^2^ of the searchable area in the infected zone were searched. Searches did not take place every day. The number of people participating on any given search day varied between two and almost 400, with more people participating at weekends. Based on the number of search participants that registered to get reimbursed, an average of 0.7 km^2^ was covered per person per day (min = 0.15 km^2^, max = 2.4 km^2^, median = 0.58 km^2^). As of 6 March 2024, 93 carcasses had been found in the area encompassing the initial infected zone.

### 4.2. Surveillance and Laboratory Analysis

Due to the advanced degree of decay of most of the carcasses, the majority of samples taken from the carcasses were bone marrow from long bones. From hunted wild boar, wild boar killed by traffic and domestic pigs, a spleen sample was the sample of choice.

In total, 62 (67%) of the 93 wild boar carcasses found in the area encompassing the initial infected zone up to 6 March 2024 tested positive for ASFV (Table 2). All ASFV-positive carcasses were found in a limited part of the core area of the outbreak, with the longest distance between two ASFV-positive carcasses being 5.1 km (Figure 2).

The sequencing of the ASFV-positive sample generated 7,136,433 paired-end reads. The sequence analyses of the six variable regions (CVR, IGR I73R/I329L, O174L, K145R, IGR MGF5059R/10R, and ECO2) of the ASFV genome revealed that the virus belonged to genotype II, genetic group 19 [26]. Genetic group 19 has been reported in nine European countries, including five EU member states. This genogroup was first detected in Romania in 2018 and has since spread west, with the last incursion in a European country being reported in Italy in January 2022 and in Croatia and Bosnia in 2023.

### 4.3. Spatiotemporal Epidemiology of the Outbreak

Using the taphonomy model, the selected carcass was assessed to have a total body score of 26. This corresponded to 1,452 ± 388 accumulated daily degrees needed to reach the score and condition of the carcass. Based on the above, the time of death of the selected wild boar was estimated to be between 66 and 117 days before it was found, i.e., sometime between 8 May and 28 June 2023. This time interval gives an indication of how long the infection may have been present in the area.

Out of the carcasses included in the pathologic photographic evaluation (*n* = 62), 12 were excluded as they were composed of incomplete sets of scattered bones (Table 2 and Figures 4(a), 4(b), 4(c), 4(d), 4(e), and 4(f)). The established categories are broad and based on several decomposition features of a carcass, hence singular skeletal fragments cannot be assessed for a post-mortem interval using photographs. For the remaining 50 carcasses, five were fresh, two had early decay, four were bloated, 17 were in the post-bloated stage, and 14 were in the late stages of decay. The animals were all estimated to have died between early May and late September, supporting the indicated time interval for the introduction of the infection based on the taphonomy model. For wild boar carcasses where a relatively long time had passed between the estimated time of death and the finding of the carcass, the span of the estimated time of the death interval is fairly long, i.e., the uncertainty of the estimate is large. For carcasses that were found relatively close to the estimated time of death, the span of the estimated time of death interval is short, i.e., the uncertainty of the estimate is small (Figure 5).

The empirical cumulative epidemic curve shows that the epidemic peak, expressed as the time with the greatest epidemic growth and visualised as the highest slope of the curve, occurred between mid-August and mid-September (Figure 5).

**Figure 6:**
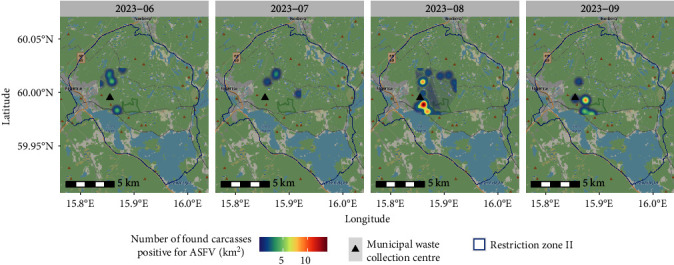
Two-dimensional kernel density estimates showing the monthly spatiotemporal evolution of the African swine fever outbreak in Sweden in 2023. The mean time of death of positive cases is displayed with the number of cases found indicated according to a heat map colour scheme. The waste collection centre is represented by a triangular shape.

The spatiotemporal analysis shows hotspot areas of the mean time of death of positive cases beginning in an area approximately 2 km north and south, respectively, of the waste collection centre during the first month of the (as yet undetected) outbreak. In the next few months, the hotspot areas of the mean time of death of positive cases gradually concentrate to the south, and a strong cluster is formed in an area approximately 1 km south of the waste collection centre in August (Figure 6).

### 4.4. Wild Boar Population and Management

**Figure 7:**
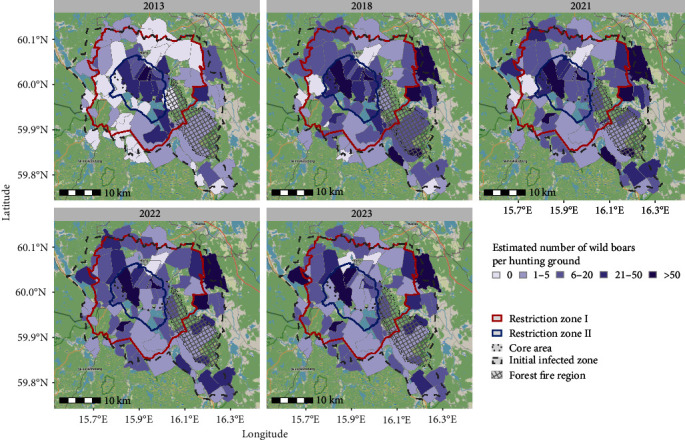
Maps indicating hunting grounds that responded to a questionnaire within the initial infected zone of the African swine fever outbreak in Sweden in 2023. The hunting ground outlines were drawn by the respondents and might not exactly reflect reality. The dashed line marks the infected zone defined on 7 September 2023, the dotted line shows the preliminary core area of outbreak, and the red and blue lines show the restricted zones established by the European Commission on 30 November 2023 (red line = restricted zone I, blue line = restricted zone II, fenced off). The respondents' estimated number of wild boar per hunting ground in 2013, 2018, 2021, 2022, and 2023 are represented by different colours.

The questionnaire was answered by 62 respondents, each representing one hunting ground within the initial infected zone, giving a response rate on hunting ground level of 65%. Sixty of the respondents (63% of hunting grounds) provided a map of the area used as their hunting ground. All hunting grounds within the core area were represented among the respondents (Figure 7). The areas used by the responding hunting grounds were between 57 and 7,738 hectares (ha) large, with an average size of 1,335 ha, and are almost exclusively covered by forests. The estimated total of man-hours used for all sorts of hunting on a hunting ground in a year varied between 50 and 3,600 hr, with an average of 817 hr and the number of hours increasing with the size of the hunting ground. Moreover, the results from the questionnaires suggest a general increase in the local wild boar population since 2013, with a notable heterogeneous distribution of wild boar between hunting grounds, and relatively high numbers of wild boar reported near the epicentre of the outbreak (Figure 7). The mean and median of the hunting bag reported in the questionnaire remained relatively stable, but with large variations between hunting grounds and a large increase in hunting grounds that shot at least one wild boar between 2013 (17 hunting grounds) and 2018 (42 hunting grounds) (Table 3). Very limited seasonal variations in the estimated numbers of wild boar by hunting ground could be seen (Supplementary Materials). According to the results from the questionnaires, baiting was frequently used and was shown to increase hunting success.

## 5. Discussion

The geographic location of Sweden, in which most areas containing wild boar populations are surrounded by water, prevents direct contact between Swedish wild boar and ASFV-infected populations in neighbouring countries. The only area that has a wild boar population and a land border is the western part of Sweden, which borders Norway. Norway has a very limited wild boar population, which is free of ASF. Disease introduction through natural wild boar movements was thus excluded. It was therefore assumed that the introduction occurred by discarded remains of virus-contaminated meat (originating from domestic pigs or wild boar in an affected country) ending up in the environment and being available to local wild boar. Although the origin of the outbreak remains unknown, it can be assumed that the long-distance translocation of the virus occurred through human activities. Similarly, the exact point of introduction cannot be established, but the municipal waste collection centre is near the outbreak epicentre and apparently contributed to the spread of ASF in the wild boar around it once the virus had been introduced into the population.

In the described outbreak, the epidemic curve seems to have peaked sometime between mid-August and mid-September, i.e., around the time of detection of the outbreak, and the last death is estimated to have occurred in late September. If the least conservative (earliest) time for the estimated first death is used, the duration of the active phase of the outbreak was thus less than 5 months. Several factors might have contributed to a favourable local epidemiology limiting disease spread and facilitating control efforts. First, the modality of the introduction: a human-mediated point introduction. This is to be compared with more challenging forms of introduction, such as those forming a constant disease pressure over a national border, for example, with an endemically infected wild boar population on the other side of the border, such as in the outbreaks in the Baltic states and Germany [2, 30, 31]; second, the relatively low wild boar density surrounding the outbreak location; and third, the location of the outbreak at the northern limit of the Swedish wild boar population range, in the northern bioregion for the species [12]. Lastly, the low availability of natural feed for wild boar in the area for large parts of the year could have facilitated control efforts, as the population could be enticed to stay in the core area by generous baiting/feeding and leaving crop fields unharvested before the fence was erected [32, 33]. Later, as the culling started, baited traps could be used to attract the remaining wild boar. The presence of natural and artificial borders (waterways, roads, railways, and road game fences) facilitated the management of the wild boar population before the fence was installed. As shown by the questionnaire data, until about 10 years ago wild boar had been completely absent from many hunting grounds in the outbreak area. At the same time, a relatively high wild boar abundance in all seasons of the year was reported from some hunting grounds, notably close to what became the epicentre of the outbreak. The municipal waste collection centre probably played a role in the pre-outbreak population dynamics and space use, serving as a year-round source of feed and with high local abundance of wild boar reported at the site. In this regard, it should be noted that a wild boar home range in this area is much larger than the hunting grounds [34], so what is shown by the wild boar abundance data is not wild boar density, but the local habitat preference of groups of wild boar.

The outbreak data demonstrate the need for high geographical resolution in risk modelling and mapping for such tools to be useful in contingency planning [35], as well as the importance of reliable and detailed wild boar population data [11]. The risk mapping undertaken in Sweden prior to the outbreak showed the municipality of Fagersta as having a relatively low risk of ASF outbreaks in wild boar, based mainly on the low average wild boar density in the area and the absence of other risk factors included in the modelling (e.g., main highways and ports). Storage of waste without wild boar-proof fencing was not a risk factor included in that model. The local wild boar abundance and space use in the hunting grounds near the epicentre of the outbreak were apparently sufficiently high to initiate and maintain the outbreak. This emphasises the localness of risk calculations and wild boar density dependency in ASF epidemiology [36, 37, 38].

Early detection followed by quick and appropriate action has been mentioned as a key factor in controlling outbreaks of ASF in wild boar in countries or areas that have been free of ASF [39]. The most effective surveillance component to achieve early detection of ASF in wild boar is passive surveillance in the form of sampling and testing of wild boar found dead [40]. Finding dead wild boar presents a challenge; however [41], sick wild boar hide and carcasses from single dead wild boar will not always be found, especially outside the hunting season when hunters and hunting dogs are less present in the forests. In the described outbreak, the epidemic growth (number of carcasses) was according to the accumulated empirical cumulative distribution of the estimated times of death slow at the start (before detection) of the outbreak. This is to be expected as ASF is of low contagiousness at low virus levels and low animal abundance [38]. In summertime, carcasses will decompose and the virus will be inactivated within weeks to months [22, 42, 43] further slowing down the epidemic growth, number of dead wild boar, and the probability of carcass finding in the beginning of an outbreak. Carcass location specificities related to easy access for forest visitors, such as proximity to paths and open or cleared areas, have been recognised as being correlated with higher finding rates [44]. Different ways to increase carcass finding rates, such as a finder's fee, active searching with dogs or drones [28, 45, 46] and searching specific habitats known as preferred deathbed choices have been studied in experimental conditions and tested during outbreaks [47, 48], but to the authors' knowledge have never been applied in ASF-free countries in pre-outbreak situations. In the described outbreak, the estimated time of death of the first carcass finds was assessed to be between 2 and 4 months before detection and reporting (with a large uncertainty in the estimate). The estimated time of death from the photographic evaluation model corresponds well with the estimated time of death from the more advanced taphonomy model. The estimated time of the first deaths is further supported by data from previously infected countries in the EU, which indicates that the infection can spread 1−2 km per month in continuous wild boar populations [20]. The maximum distance between positive cases in the described outbreak was 5.1 km, reached already in the second week after the outbreak was detected. In accordance with the two pathologic evaluations, this also indicates the presence of infection for approximately two to 4 months prior to detection [20, 49]. It might, however, be argued that this figure is not relevant for this outbreak as the population is not continuous and the outbreak area is much smaller than those of outbreaks that have been used to estimate the average speed of the spread of ASF in outbreaks in the EU [16]. Moreover, the heterogeneous use of the habitat and low wild boar density in the area might reduce the speed of spread as wild boar do not frequently move through large areas of unfavourable habitats [34]. Finally, the local forests with dense vegetation, offering plenty of hiding spaces for sick wild boar, and terrain not attractive or easily available for dog walkers and leisure forest activities, as well as the season with few hunters in the forests during the first months after the presumed time of introduction (the main hunting season in Sweden is during autumn and winter), could explain the observed time interval between death and detection of the first carcasses.

The set of measures available for control of ASF in wild boar is limited and comprises carcass removal, fencing, restrictions on public access, targeted feeding, and culling [9]. The local epidemiology described in the previous sections contributed to that eradication, which could be positively achieved using these available tools. Culling started when the core area was fenced, at a time when few wild boar remained and there was no longer any ongoing spread of the disease. All culled wild boar tested negative for ASF. The impact that culling had on the eradication of the outbreak is open to debate. As the survival of ASFV in the environment during Nordic winters is not entirely known [43], it was considered important not to have any remaining wild boar in the core area, and specifically not to have wild boar reproducing, after the winter. This would have complicated surveillance and prolonged the process to regain freedom from ASF. Another issue supporting culling at the current point in time was a concern that wild boar movement and thus disease spread could be triggered by the rutting season that starts in November [50, 51]. The ban on access to the infected zone was considered an important control measure, especially at the beginning of the outbreak before the level of contamination in the environment or the geographical extension of the outbreak was known. As it became obvious that the outbreak was limited to a small part of the core area, access restrictions were progressively lifted. Despite this, the negative impact of the restrictions on the people living in the area and on local and regional business, tourism, and industries was substantial (see, e.g., https://www.skogsaktuellt.se/artikel/2234210/nstan-varje-dag-fr-jag-en-ny-infallsvinkel-p-hur-det-hr-slr.html). SBA did random checks on restriction compliance and no major breaches were found. The goodwill and collaboration of groups of people affected by the restrictions were important factors in the control, as has been discussed in previous research [19, 52, 53]. The existing compensation schemes for regulated diseases in Sweden include losses for farmers affected by the diseases or restrictions, but not stakeholders and third parties such as forestry and tourism. Lack of compensation may affect trust in authorities and future compliance with restrictions.

The competent authorities in Sweden could benefit from the experiences of previously infected countries, past networking efforts and capacity building, as well as contingency planning and outbreak exercises performed prior to the outbreak [54]. Nevertheless, it was obvious that it is not possible to prepare for or anticipate everything and that, for example, the practical solutions for carcass search, sampling, and testing logistics have to be adapted to local circumstances and evolve during the outbreak. In this regard, it was deemed very positive how existing mobile applications and data sources could be combined with custom-made applications and systems for information retrieval and sharing, for supporting central decision-making and for use as feedback to the field for planning carcass searches. In this case, it was partly made possible by the collaboration developed with a private company (WeHunt®) and partly by the current favourable conditions for data collection and communication where advanced modern technology can be used effectively by laymen and in tough conditions [55]. The pre-outbreak preparatory work included various communication campaigns aimed at reducing identified risks of introduction of ASF (e.g. waste in roadside rest areas and camping sites). In this regard, the risk-assessment and risk-handling authorities (SVA and SBA, respectively) had not identified municipal waste collection centres as a specific risk. According to existing national and EU regulations (Directive (EU) 2018/851), these sites should not endanger human, animal, or environmental health (although contagious diseases are not specifically mentioned), something that was found not to be properly implemented in this case. Considering the long-distance translocation of ASFV that has occurred during the current epidemic in Europe, preventing wild boar access during all steps in the handling and storage of waste should remain a high priority for ASF-free countries.

## 6. Conclusions

In summary, the outbreak of ASF in wild boar in Sweden seems well on the way to becoming one of so far only three successful eradication campaigns in Europe during the current global ASF pandemic (together with those in Czech Republic and Belgium) [10, 56]. Some, but not all, of the reasons for these three separate accomplishments are known. In the outbreak described in this paper, the local epidemiology seems to have been one factor that facilitated its control. In addition, the multi-stakeholder collaboration that was set up prior to the outbreak was an invaluable asset, as was the involvement of SJF including the use of its databases and knowledge. Engagement on a regional and local level could boost and make use of the commitment of local hunters for searches and culls and avoid the kind of conflicts that have been seen in other affected countries [57].

## Figures and Tables

**Table 1:** Estimated time interval (days between the time of death and the time of finding the carcass) for each of the six stages of decomposition, based on Rietz et al. [28].

Time	Stage	Description	Figure
0–1 days	Fresh	Cold carcass but no signs of decay or no severe smell	4a
2–6 days	Early decay	Leakage of bodily fluids, discolouration of the skin	4b
7–14 days	Bloated	Carcass bloated, loss of skin, and hair due to decay	4c
2–4 weeks	Post-bloated	Abdominal cavity open, organs reduced to a fluid, bodily fluids distributed to the surrounding grounds, loss of musculature due to decay	4d
1.5–4 months	Advanced decay	Carcass left by insects, visible bones, remaining skin is dry, and mummified or putrefied	4e
>5 months	Dry remains	Only skeletal remains, with minor dry skin remains, possibly with moss or algae growth on bones	4f

**Table 2:** Test results for African swine fever in the outbreak in Sweden from 6 September 2023 to 6 March 2024.

Sample type/area	Number of ASFV-positive samples	Number of ASFV-negative samples	Total number of samples
Infected zone/restricted zones ^*∗*^			
Wild boar carcasses	62	31	93
Wild boar killed by traffic	0	8	8
Culled wild boar	0	84	84
Enhanced surveillance area ^*∗*^			
Hunted wild boar	0	75	75
Wild boar killed by traffic	0	3	3
Wild boar carcasses	0	4	4
Domestic pigs	0	24	24

^*∗*^The infected zone was replaced with restricted zones on 30 November 2023 (Figure 2). The enhanced surveillance area encompassed all the municipalities surrounding the infected zone and changed accordingly. The results refer to samples taken within the infected zone/restricted zones or surveillance area at any given date. ASFV, African swine fever virus.

**Table 3:** The wild boar hunting bag of the hunting grounds where, according to the questionnaire, respondents (*n* = 17–46) shot at least one wild boar during the year in question, from an online questionnaire completed during the outbreak of African swine fever in Sweden in 2023.

Year	Hunting grounds ^*∗*^	Total number of shot wb (per 100 ha)	Min	Max	Median	Mean	Lower CI	Upper CI
2013	17	125 (0.16)	1	45	5	7.35	2.06	12.64
2018	42	350 (0.44)	1	38	5	8.33	5.73	10.93
2021	46	380 (0.48)	1	65	4	8.26	4.95	11.57
2022	46	275 (0.35)	1	30	3	5.98	3.94	8.02
2023^*∗∗*^	31	125 (0.16)	1	15	2	4.03	2.78	5.28

^*∗*^Number of hunting grounds that reported shooting at least one wild boar in that year;  ^*∗∗*^2023 encompasses 1 January to 6 September, i.e., the part of year that did not include the main hunting season. wb, wild boar; min, minimum value; max, maximum value; and CI, confidence interval.

## Data Availability

Data supporting the conclusions of the study can be accessed upon reasonable request by e-mail to the first author (Erika Chenais).

## Supplementary Materials

Supplementary Materials The supplementary materials includes timeline of the outbreak, carcass protocol, questionnaire about wild boar population and management, and maps showing the seasonal space-use of wild boar in the infected zone in year 2022 based on results from a questionnaire to leaders of hunting groups.
