# Supplementary material for: First Outbreak of African Swine Fever in Sweden: Local Epidemiology, Surveillance, and Eradication Strategies
Source: Transbound Emerg Dis. 2024 Jun 26;2024:6071781. doi: 10.1155/2024/6071781 (PMC12017073; doi:10.1155/2024/6071781)

# Supplementary materials

1. Timeline
2. Carcass protocol
3. Questionnaire
4. Maps showing the seasonal space-use of wild boar in the infected zone in year 2022 based on results from a questionnaire to leaders of hunting groups.

## TIME Line


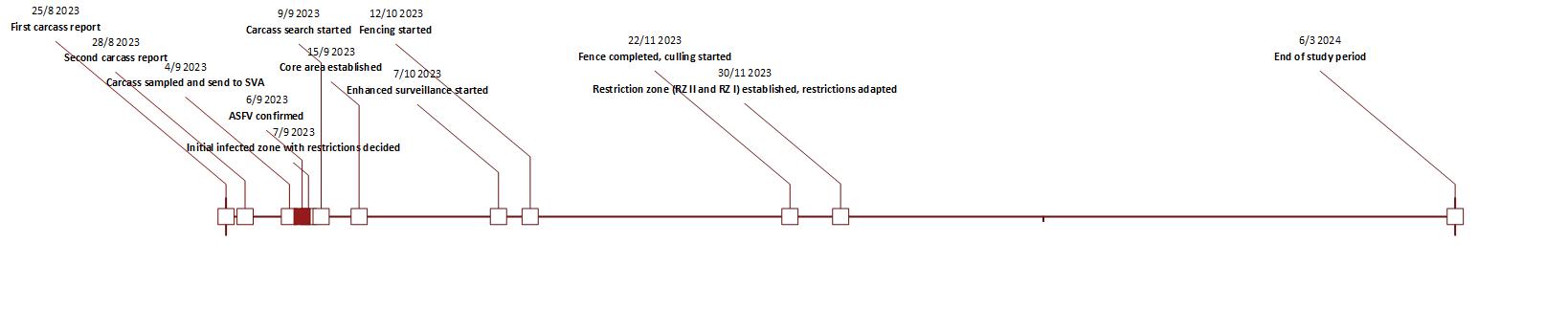


## Carcass Protocol

# Checklista vildsvinskadaver

plats för rapport-ID-nummer

(fylls i på provtagningscentral)

##

## 1. Fyndplats och upphittare

#### Obligatorisk information: Fyll i ett formulär per djur. Om flera djur påträffas ska de märkas med löpnummer (1, 2, 3, etc). Om flera djur med **samma status** hittas på **samma plats** räcker det att fylla i hela formuläret för det **första** djuret och att för övriga djur endast fylla i information under punkt 2 och att fotografera enligt instruktion. Skicka formulär och prover för alla djur från samma fyndplats tillsammans.

| Kontaktperson, namn__________________________________ Telefon___________________  Fynddatum**___________________________________________** **Fyndplatsens namn______________________________________________________________**  **Fyndplatsens** GPS koordinater X: Y: (ex: X 59.82312, Y 17.65408)  (från Google maps, dvs WGS 84 dec. i första hand, annars ange vilket system som används):_______________________________________________________________________  Typ av miljö/habitat 🞏 Åker 🞏 Skog 🞏 Vass/strand 🞏 Vatten 🞏 Annat: ___________________  Sol/skugga 🞏 Solskyddat läge 🞏 Ej solskyddat läge  **Fotografera:**   - Översiktsbild av kadavret från två olika vinklar. Använd gärna något som storleksjämförelse, i första hand måttband/linjal. - Intressanta detaljer (till exempel närbild mun/tryne, eventuella öppningar i kadavret, tydliga sjukliga förändringar) - Översiktsbild på omgivningen där kadavret hittades.   Namnge bilderna med: Datum, kontaktperson, fyndplatsens namn, djurets löpnummer.  Maila bilderna till **vilt@sva.se** |
| --- |

## 2. Vildsvin

#### Identifiering av djuret: Bedöm om möjligt vikt och kön. Om något inte går att bedöma, lämna aktuellt fält tomt.

| Djurets löpnummer (ex 1. 2. 3 osv vid flera djur) __________  Vikt på djuret 🞏 Under 30kg 🞏 30-80kg 🞏 över 80kg  Kön 🞏 Hane 🞏 Hona |
| --- |

3. bedömning av förruttnelsegraden

| **Fynd (kryssa i de rutor som stämmer)** |
| --- |
| 🞏 Kall kropp, ingen synlig nedbrytning eller lukt  🞏 Likstelhet  🞏 Kroppsvätskor läcker från tryne, mun, eller öron  🞏 Färgförändringar i hud (blågröna)  🞏 Uppsvälld  🞏 Hår eller hud som lossnar  🞏 Buken öppnad  🞏 Smältande/rinnande organ; läckage av förruttnelsevätskor ut på marken  🞏 Ihopsjunken kropp  🞏 Synliga skelettdelar men fortfarande till stor del täckt med vävnad  🞏 Mjukdelsvävnader och inre organ försvunna; kvarvarande hud/vävnad är torr, svart (mumifierad) eller något kladdig  🞏 Endast skelett med hudrester  🞏 Endast blekta skelettdelar kvar. Mossa, alger eller svamp kan växa på vävnaderna |

### 4. Aktivitet av insekter

 Ingen

 Flugägg men inga larver

 Ringa larvaktivitet

 Riklig larvaktivitet

 Spridning av larver till omkringliggande omgivning

### 5. Spår efter asätare

 Inga spår

 Asätare har hackat på eller ätit av ögon och eller nos/anus

 Asätare har hackat på eller ätit på/ur buken

 Asätare har tuggat på kadavrets ben eller slitit av dem

 Kroppsdelarn

## Questionnaire

### Outbreak of ASF in wild boar

SVA and the Swedish Hunters' Association want to use this survey to increase knowledge about wild boar in the infected zone. The questionnaire is addressed to all hunting leaders within the infected zone and consists of two parts, both equally important:

1) this survey

2) to send in a map file with the hunting ground that this questionnaire refers to marked by e-mail to stefan.widgren@sva.se. Also name the hunting ground in the email. The best is if you can send a map as a gpx file. If sending a gpx file doesn't work, you can send a screenshot or a photo of a map.

The survey consists of 12 questions and is expected to take 10-15 minutes to complete.

**1) Your contact details:**

Name:

E-mail:

Phone number:

**2) Enter the name of the hunting ground where you are the hunting leader. It is this hunting ground that the rest of the survey is about:**

**3) How big is the hunting ground (hectares)?**

**4) Which habitat dominates the hunting ground?**

Forest:

Agricultural lands and fields:

Other:

**5) Approximately how many man-hours are spent by the hunting team on all kinds of hunting per year on the hunting grounds during a normal year?**

**6) How many wild boar do you estimate to have been the most on the hunting ground in the last ten years?**

|  | None | 1-5 | 6-20 | 21-50 | >50 |
| --- | --- | --- | --- | --- | --- |
| 2023 |  |  |  |  |  |
| 2022 |  |  |  |  |  |
| 2021 |  |  |  |  |  |
| 2018 (five years ago) |  |  |  |  |  |
| 2013 (ten years ago) |  |  |  |  |  |

**7) In 2022, how many wild boar did you experience on the hunting ground during different parts of the year?**

Winter: January-March

Spring and early summer: April-June

Summer and early autumn: July-September

Autumn and winter: October-December

**8) How many wild boar were shot on the hunting ground these years (if the exact number is unknown, give an estimate)?**

2023

2022

2021

2018 (five years ago)

2013 (ten years ago)

**9) Other comments about the presence of wild boar or development of the wild boar population in the area:**

**10) Are there or have there been wild boar baiting stations on the hunting ground in the last ten years?**

|  | No | 1 | 2 | 3 or more |
| --- | --- | --- | --- | --- |
| 2023 |  |  |  |  |
| 2022 |  |  |  |  |
| 2021 |  |  |  |  |
| 2018 (five years ago) |  |  |  |  |
| 2013 (ten years ago) |  |  |  |  |

**11) Are there game cameras on the hunting grounds now?**

No

Yes, 1

Yes, 2

Yes, 3 or more

**12) Other comments or views:**

## WIldboar Seasonal space-use in the infected zone in year 2022


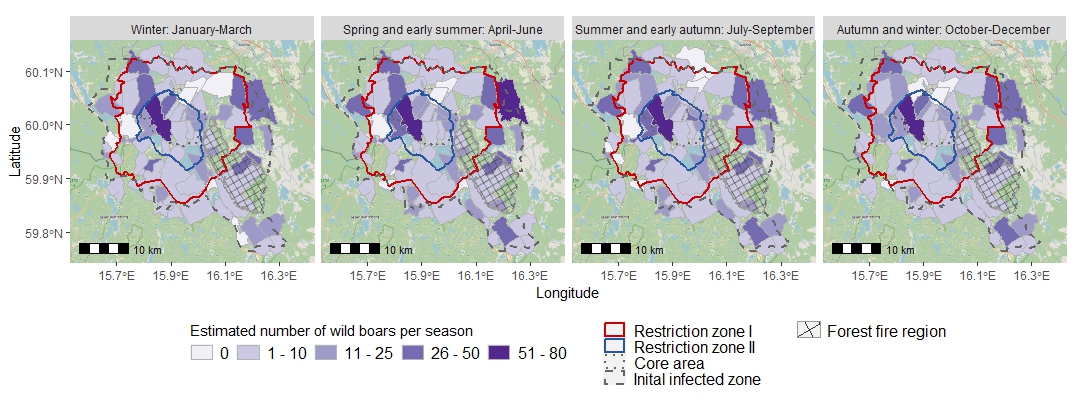

Supplement: Supplementary Materials — The supplementary materials includes timeline of the outbreak, carcass protocol, questionnaire about wild boar population and management, and maps showing the seasonal space-use of wild boar in the infected zone in year 2022 based on results from a questionnaire to leaders of hunting groups. [file 6071781.f1.docx]
